# Supplementary figures and images for: Endogenous production of hyaluronan, PRG4, and cytokines is sensitive to cyclic loading in synoviocytes
Source: PLoS One. 2022 Dec 28;17(12):e0267921. doi: 10.1371/journal.pone.0267921 (PMC9797074; doi:10.1371/journal.pone.0267921)

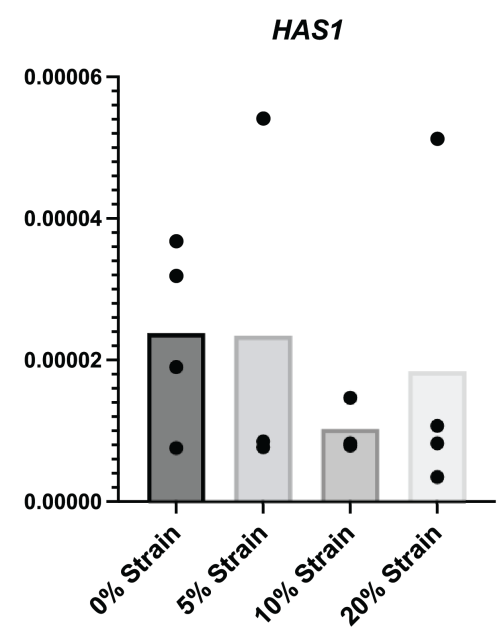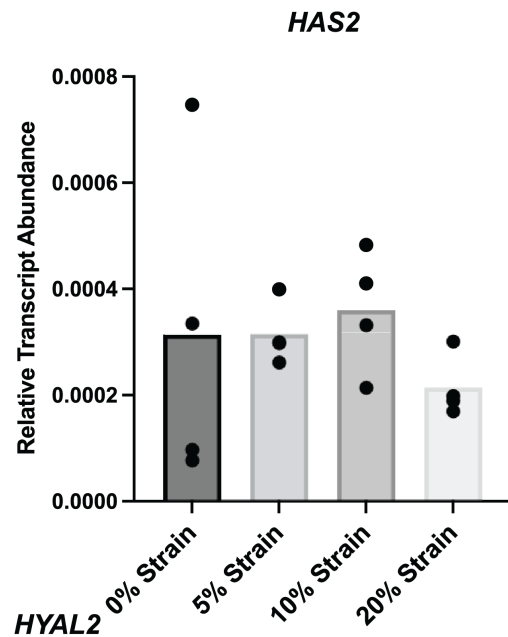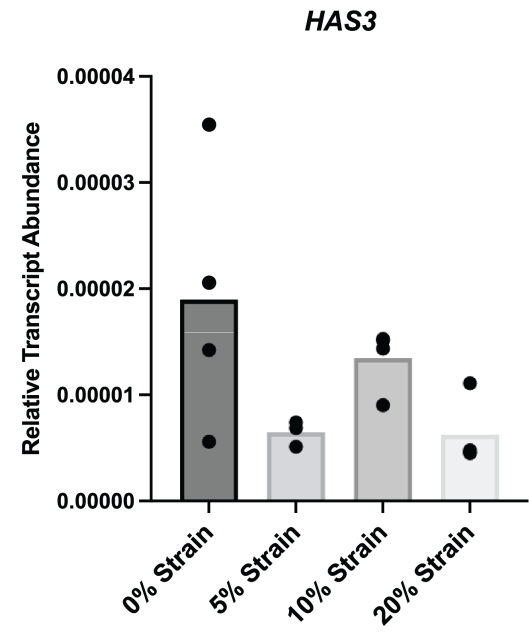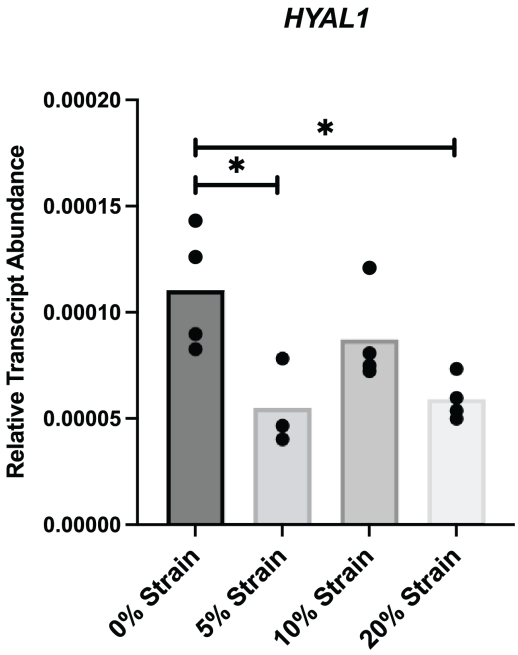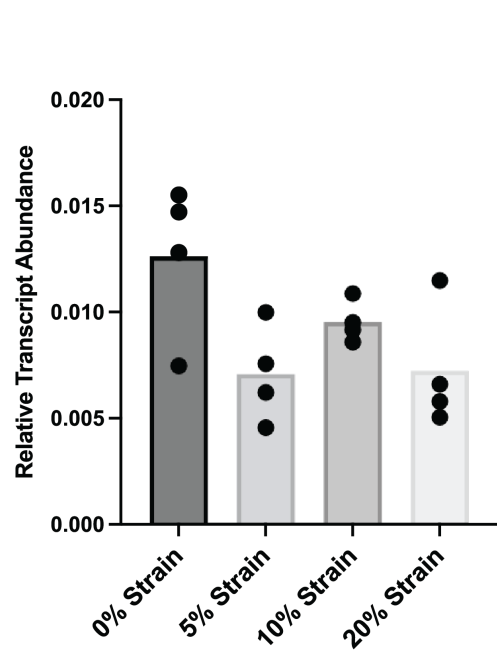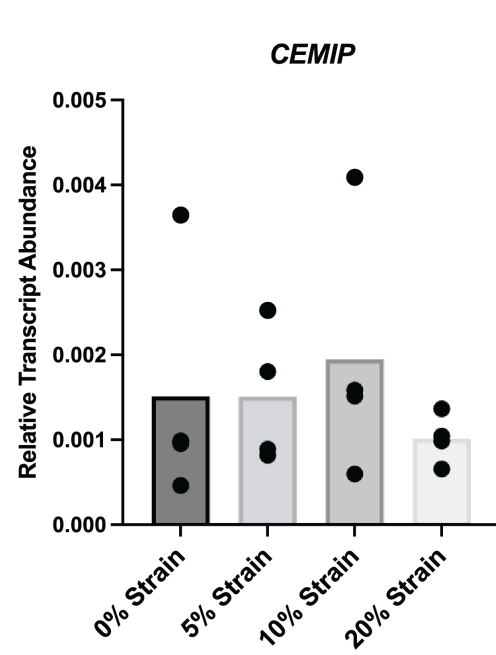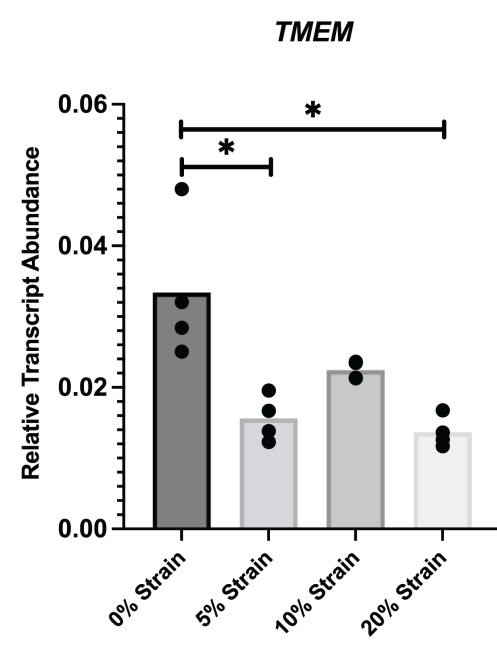

Supplement: S1 Fig — Black circles represent individual samples with significant differences between groups are shown with brackets. (PDF) [file pone.0267921.s001.pdf]

# PRG4

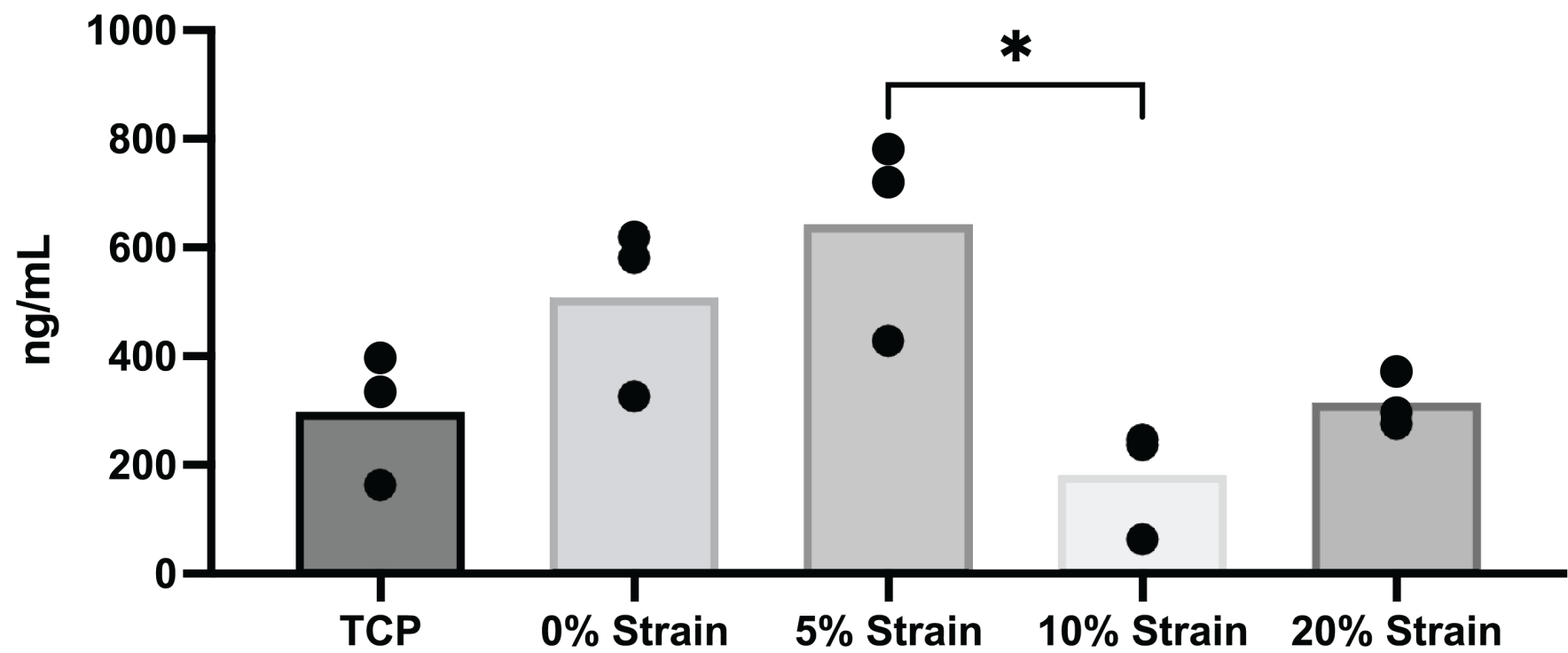

Supplement: S2 Fig — Black circles represent individual samples with significant differences between groups are shown with brackets. (PDF) [file pone.0267921.s002.pdf]

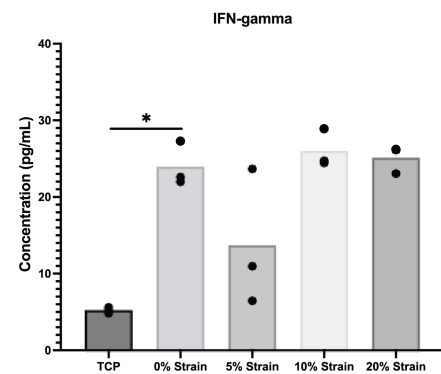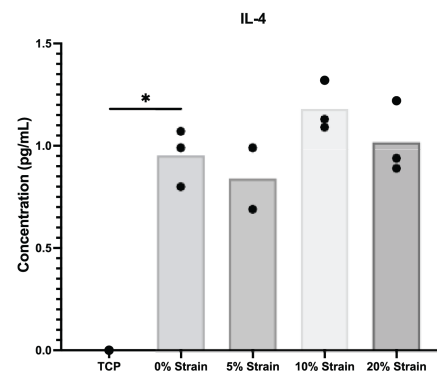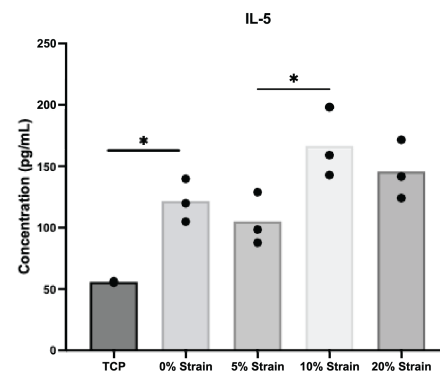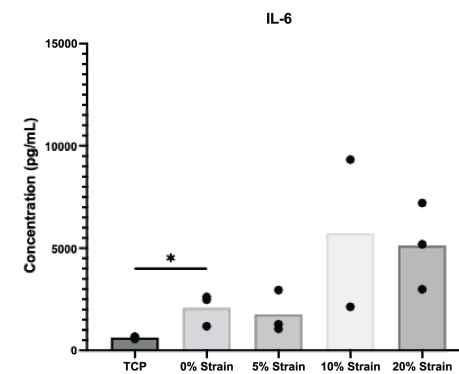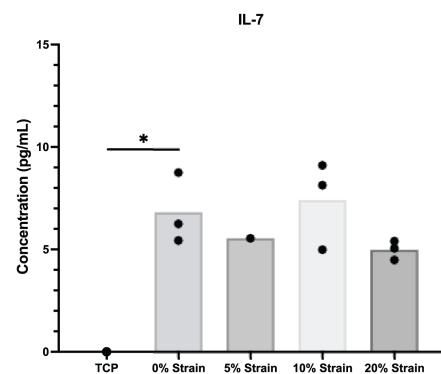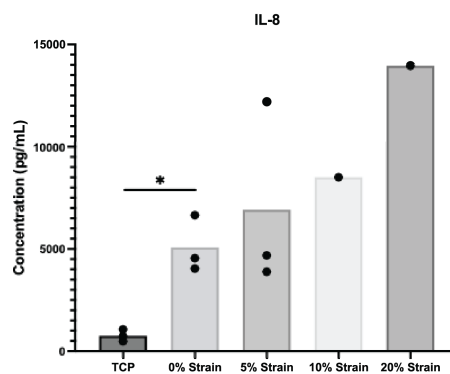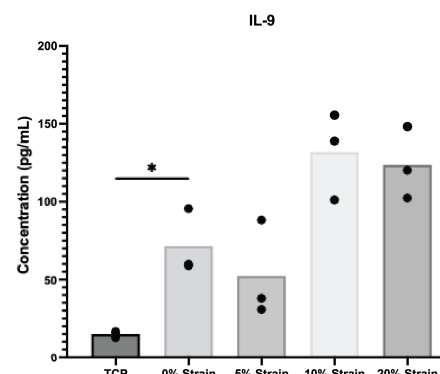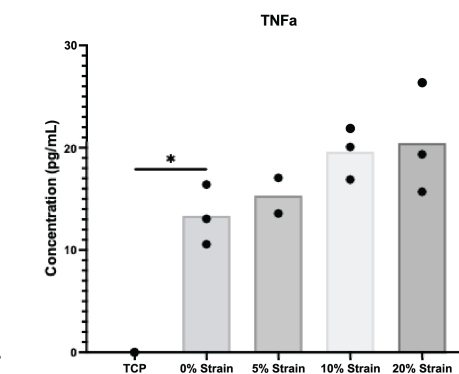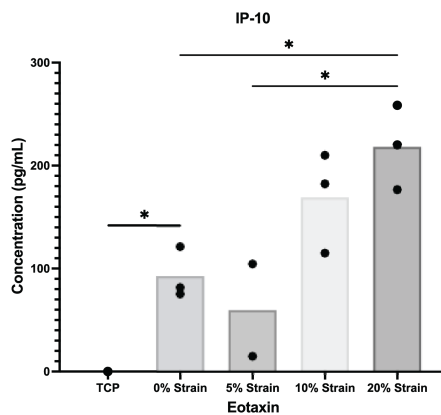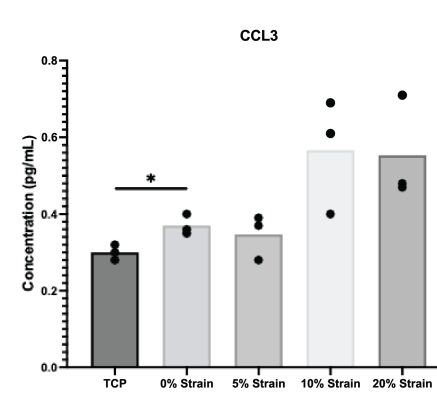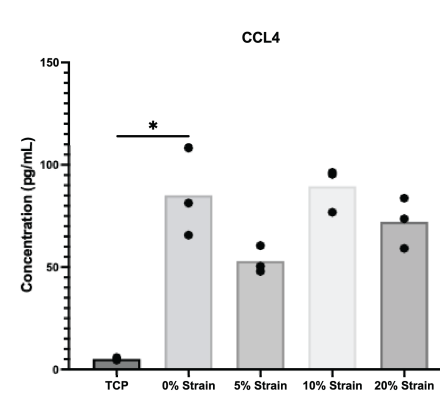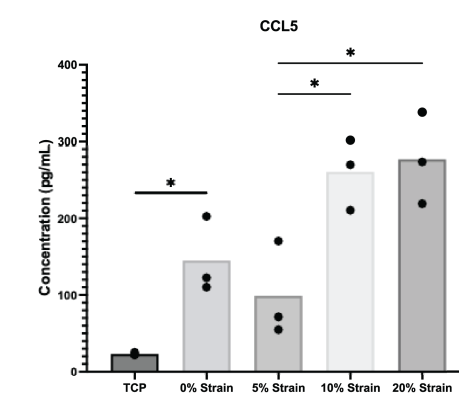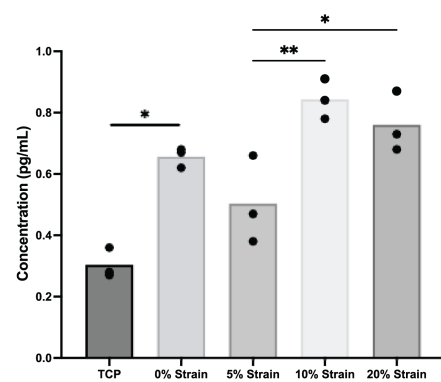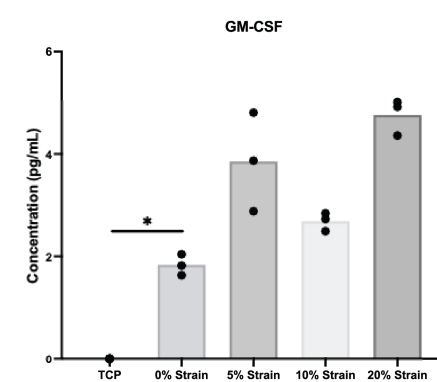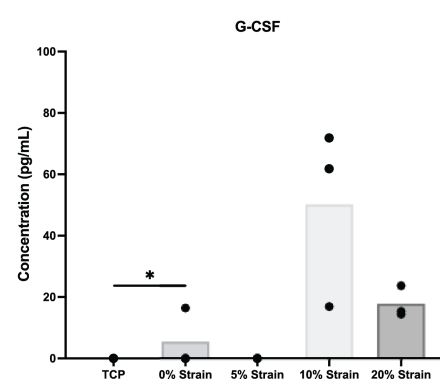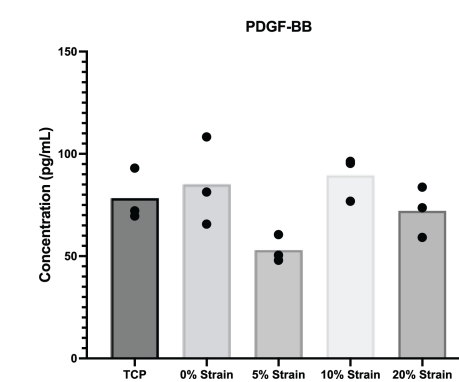

Supplement: S3 Fig — Black circles represent individual samples with significant differences between groups are shown with brackets. Significance between the TCP and 0% strain group was established using a t test or non-parametric alternative. Significance between the 0%, 5%, 10%, and 20% strain groups was established using a one-way ANOVA or non-parametric alternative. Concentrations are shown as pg/mL. (PDF) [file pone.0267921.s003.pdf]

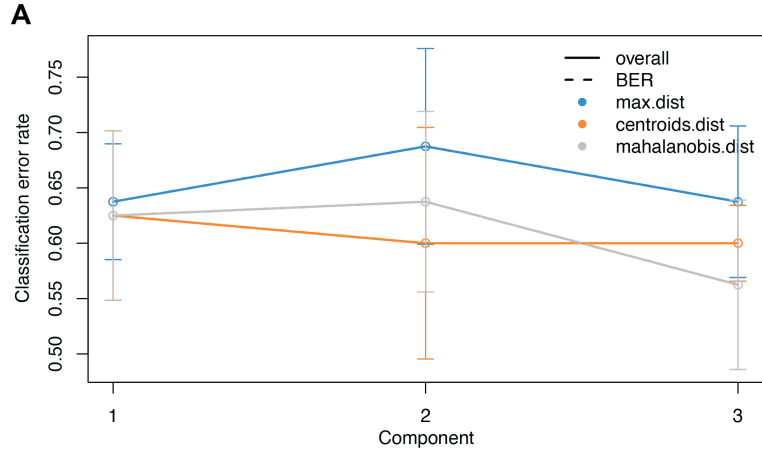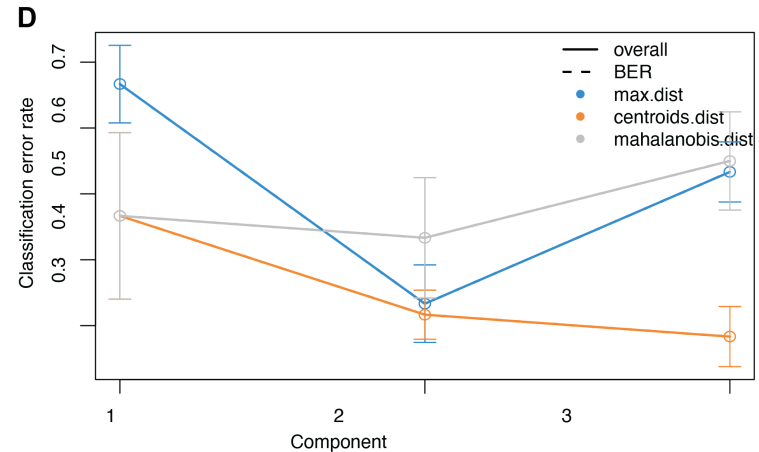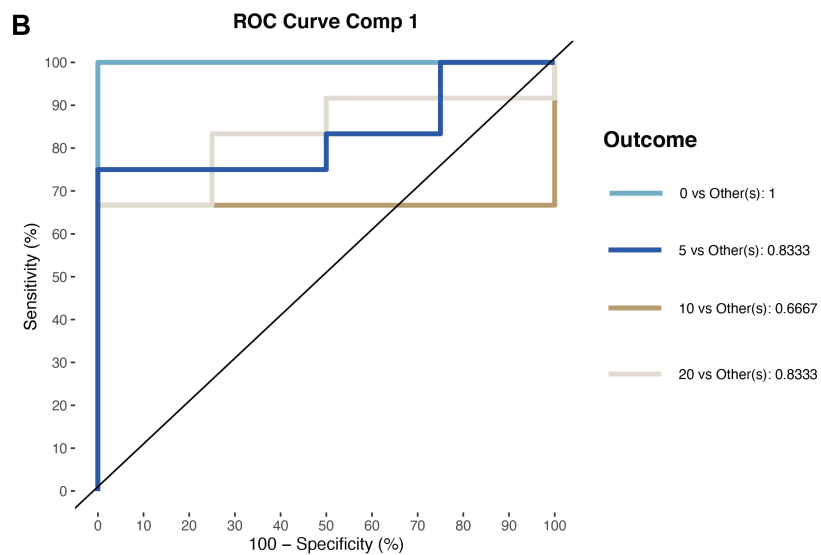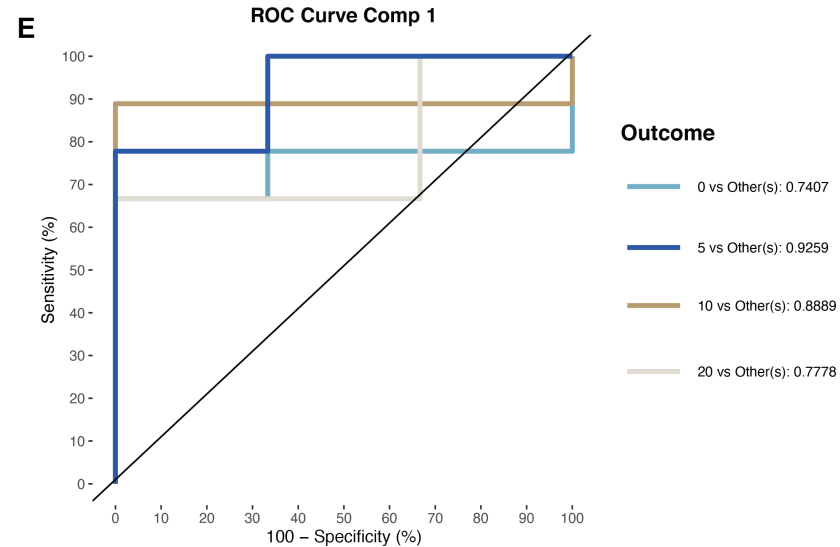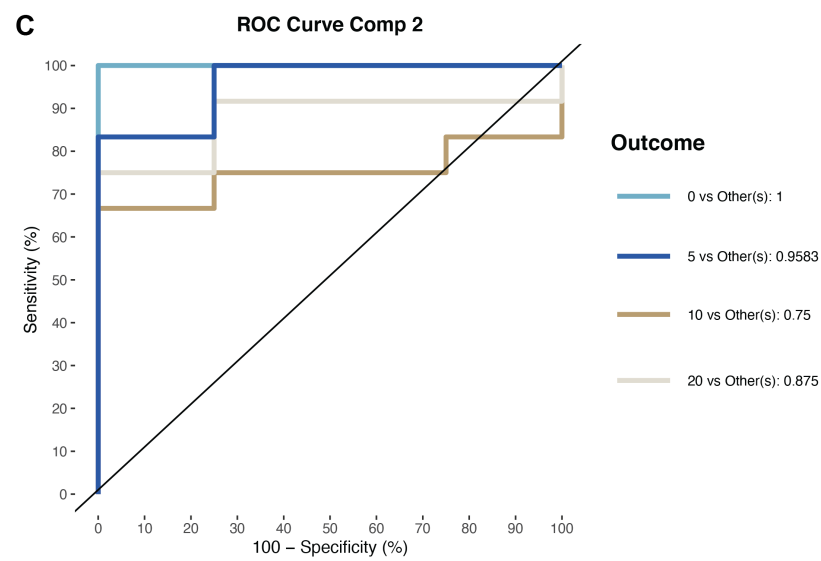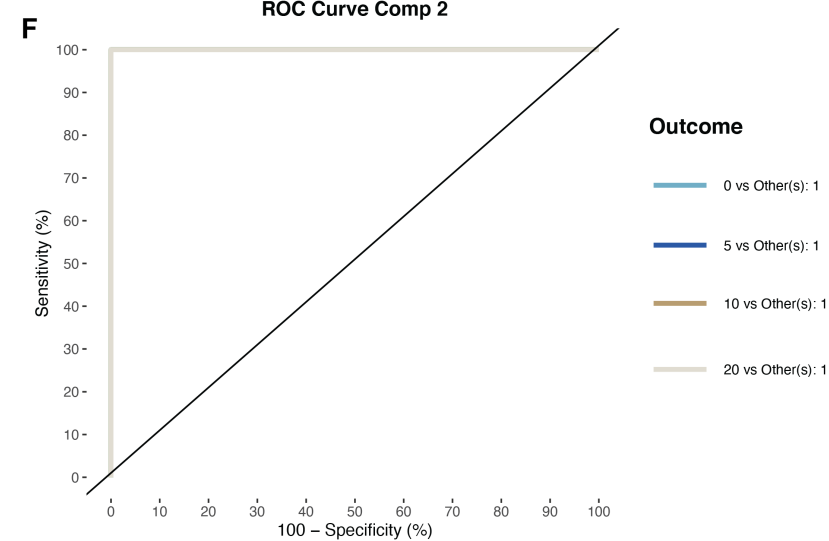

Supplement: S4 Fig — Classification error rates and ROC curves of each component for A) gene expression and B) cytokine concentration predicting strain group. (PDF) [file pone.0267921.s004.pdf]
