# Supplementary material for: Endogenous production of hyaluronan, PRG4, and cytokines is sensitive to cyclic loading in synoviocytes
Source: PLoS One. 2022 Dec 28;17(12):e0267921. doi: 10.1371/journal.pone.0267921 (PMC9797074; doi:10.1371/journal.pone.0267921)
Supplement: S5 Fig — Q2total values of each component for A) gene expression predicting hyaluronan concentration, B) gene expression predicting PRG4 concentration, C) cytokine concentration predicting hyaluronan concentration, and D) cytokine concentration prediction PRG4 concentration. (PDF) [file pone.0267921.s005.pdf]

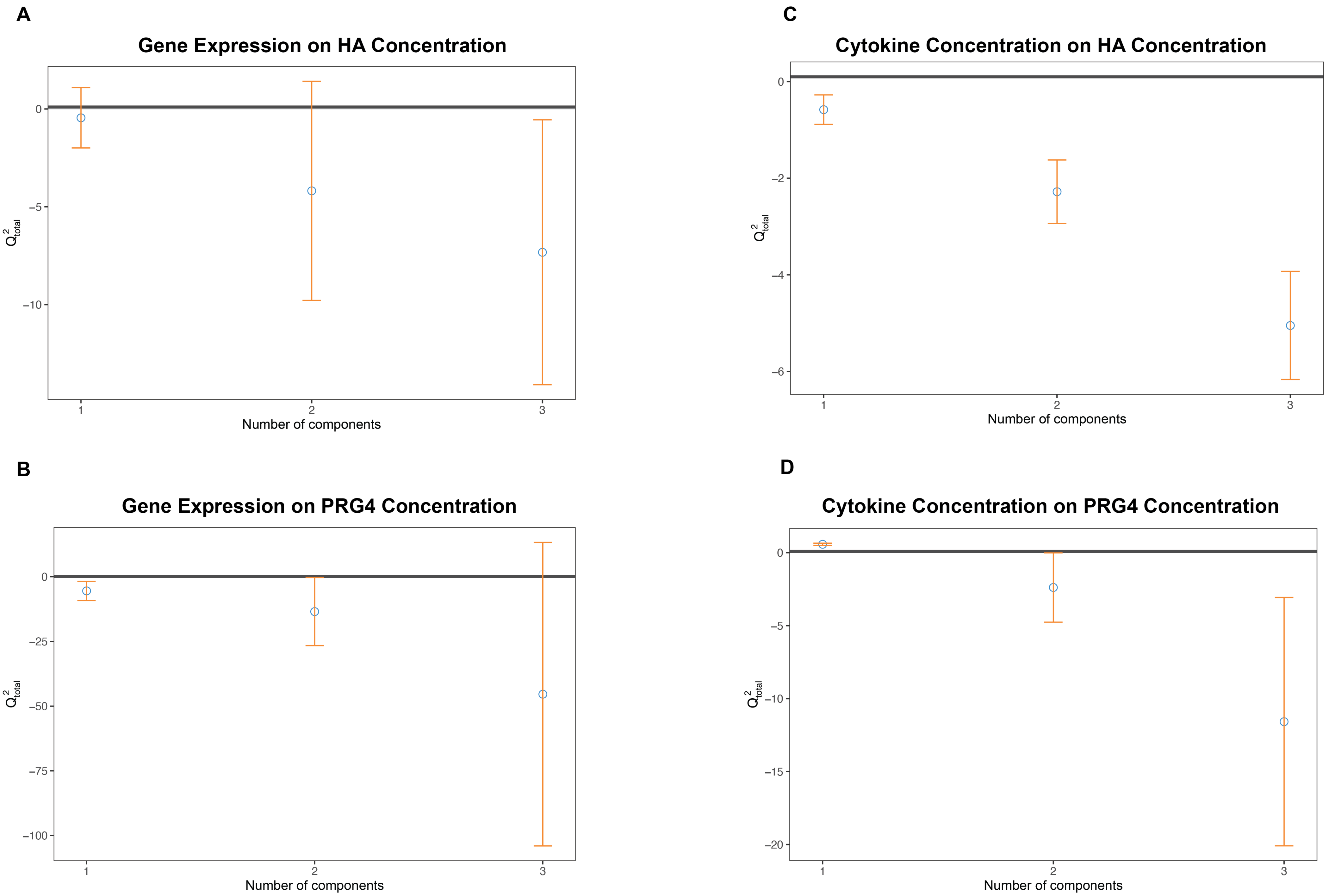

A
Gene Expression on HA Concentration
0
−5
−10
Q^2_{total}
1
2
3
Number of components
B
Gene Expression on PRG4 Concentration
0
−25
−50
−75
−100
Q^2_{total}
1
2
3
Number of components
C
Cytokine Concentration on HA Concentration
0
−2
−4
−6
Q^2_{total}
1
2
3
Number of components
D
Cytokine Concentration on PRG4 Concentration
0
−5
−10
−15
−20
Q^2_{total}
1
2
3
Number of components
