# Supplementary material for: Endogenous production of hyaluronan, PRG4, and cytokines is sensitive to cyclic loading in synoviocytes
Source: PLoS One. 2022 Dec 28;17(12):e0267921. doi: 10.1371/journal.pone.0267921 (PMC9797074; doi:10.1371/journal.pone.0267921)
Supplement: S1 Table — (PDF) [file pone.0267921.s006.pdf]

Gene Symbol	Gene	Inventory Number
HAS1	Hyaluronan Synthase 1	Hs00758053_m1
HAS2	Hyaluronan Synthase 2	Hs00193435_m1
HAS3	Hyaluronan Synthase 3	Hs00193436_m1
HYAL1	Hyaluronidase 1	Hs00201046_m1
HYAL2	Hyaluronidase 2	Hs00186841_m1
CEMIP	Cell Migration Inducing Hyaluronidase	Hs01552114_m1
TMEM	Cell Surface Hyaluronidase	Hs00910521_m1
GAPDH	glyceraldehyde-3-phosphate dehydrogenase	Hs02786624_g1
