# Supplementary material for: Endogenous production of hyaluronan, PRG4, and cytokines is sensitive to cyclic loading in synoviocytes
Source: PLoS One. 2022 Dec 28;17(12):e0267921. doi: 10.1371/journal.pone.0267921 (PMC9797074; doi:10.1371/journal.pone.0267921)
Supplement: S2 Table — (PDF) [file pone.0267921.s007.pdf]

	**Average GAPDH Cq Value**
TCP	20.493 ± 0.282
0% Strain	20.896 ± 0.376
5% Strain	22.433 ± 3.567
10% Strain	20.76 ± 0.461
20% Strain	20.651 ± 0.786
