# Supplementary material for: Endogenous production of hyaluronan, PRG4, and cytokines is sensitive to cyclic loading in synoviocytes
Source: PLoS One. 2022 Dec 28;17(12):e0267921. doi: 10.1371/journal.pone.0267921 (PMC9797074; doi:10.1371/journal.pone.0267921)
Supplement: S3 Table — (PDF) [file pone.0267921.s008.pdf]

	TCP	0% Strain	5% Strain	10% Strain	20% Strain
HAS1	37.34	36.485 ± 1.257	36.653 ± 1.401	37.458 ± 0.159	37.065 ± 1.058
HAS2	30.832 ± 0.145	33.129 ± 1.495	34.085 ± 3.623	32.265 ± 0.962	32.873 ± 0.489
HAS3	36.541 ± 0.145	36.871 ± 1.071	37.908 ± 0.388	36.974 ± 0.821	38.057 ± 0.220
HYAL1	36.038 ± 0.823	34.079 ± 0.273	34.865 ± 0.368	34.279 ± 0.122	34.713 ± 0.555
HYAL2	27.341 ± 0.197	27.259 ± 0.535	29.635 ± 3.205	27.379 ± 0.521	27.839 ± 0.379
CEMIP	28.067 ± 1.786	30.7 ± 1.182	31.97 ± 3.336	30.091 ± 0.825	30.648 ± 1.176
TMEM	31.997 ± 4.087	25.847 ± 0.405	28.46 ± 3.333	26.241 ± 0.432	26.858 ± 0.577
GAPDH	20.493 ± 0.281	20.907 ± 0.375	22.433 ± 3.566	20.761 ± 0.461	20.651 ± 0.786
