# Supplementary material for: Endogenous production of hyaluronan, PRG4, and cytokines is sensitive to cyclic loading in synoviocytes
Source: PLoS One. 2022 Dec 28;17(12):e0267921. doi: 10.1371/journal.pone.0267921 (PMC9797074; doi:10.1371/journal.pone.0267921)
Supplement: S4 Table — Zeroes indicate a value that fell below the lower limit of the standard curve. All cytokines are shown as pg/mL. (PDF) [file pone.0267921.s009.pdf]

Sample	Basic FGF	Eotaxin	G-CSF	GM-CSF	IFN-alpha	IL-4	IL-5	IL-6	IL-7	IL-8	IL-9	IP-10	MCP-1	MIP-1alpha	MIP-1beta	PDGF-BB	RANTES	TNF-alpha
TCP	0	0.27	0	0	4.84	0	0	671.81	0	481.78	16.66	0	1588.96	0.32	4.97	93.04	25.46	0
TCP	0	0.28	0	0	5.62	0	56.4	656.2	0	1063.42	15.5	0	1640.19	0.28	5.86	69.61	22.44	0
TCP	0	0.36	0	0	5.31	0	55.12	559.55	0	722	12.7	0	1471.04	0.3	4.61	72.15	22.07	0
0% Strain		0.67	0	1.63	22	0.8	104.98	1185.65	6.25	4038.46	95.51	81.69	3635.12	0.4	30.14	65.64	202.6	13.06
0% Strain	20.6	0.62	0	1.82	22.61	0.99	119.91	2615.02	8.75	4548.96	59.91	75.44	2789.46	0.36	21.87	81.33	122.52	10.57
0% Strain	21.72	0.68	16.39	2.04	27.29	1.07	139.89	2477.91	5.44	6654.59	58.85	121.45	0	0.35	20.85	108.25	110.2	16.41
5% Strain	0	0.47	0	3.87	10.98	0.69	98.48	1287.15	0	4683.47	37.97	14.92	2617.54	0.37	13.87	60.53	71.57	13.58
5% Strain	0	0.38	0	2.88	6.47		87.78	1057.62	0	3883.74	30.83		1871.55	0.28	11.44	47.96	54.92	0
5% Strain	42.62	0.66	0	4.81	23.67	0.99	128.86	2954.39	5.55	12197.95	88.17	104.72	0	0.39	30.56	50.48	170.38	17.06
10% Strain	19.67	0.84	16.89	2.73	28.9	1.09	142.87	2133.87	8.14	8510.82	155.51	115.19	2966.67	0.4	46.09	96.35	301.67	20.08
10% Strain	19.91	0.91	61.82	2.84	24.76	1.32	197.92		9.11		138.83	210.13	2940.26	0.69	46.76	95.34	269.75	21.88
10% Strain	0	0.78	71.87	2.49	24.45	1.13	159.15	9333.53	4.99		101.16	182.41	3102.27	0.61	33.24	76.83	210.56	16.9
20% Strain	0	0.68	15.31	4.92	23.07	0.94	124.12	2988.45	4.49	13962.01	102.41	176.79	0	0.48	34.19	73.67	219.03	19.35
20% Strain	39.73	0.87	23.7	5.01	26.22	1.22	171.44	7211.95	5.4		148.24	258.55	0	0.71	49.19	83.7	338.24	26.36
20% Strain	0	0.73	14.4	4.36	26.12	0.89	141.7	5189.51	5.06		120.23	220.11	0	0.47	38.73	59.15	273.58	15.69
