# Supplementary material for: Endogenous production of hyaluronan, PRG4, and cytokines is sensitive to cyclic loading in synoviocytes
Source: PLoS One. 2022 Dec 28;17(12):e0267921. doi: 10.1371/journal.pone.0267921 (PMC9797074; doi:10.1371/journal.pone.0267921)
Supplement: Attachment — Submitted filename: Response to Reviewers.pdf [file pone.0267921.s011.pdf]

PURDUE UNIVERSITY | Weldon School of Biomedical Engineering

December 8, 2022

RE: Resubmission of Revised Manuscript

To the PLoS One Editorial Staff and reviewers:

Thank you for the additional comments on our manuscript titled “Endogenous Production of Hyaluronan, PRG4, and Cytokines is Sensitive to Cyclic Loading in Synoviocytes.” Each point has been addressed and changes to the original manuscript are in a Track Changes version. To easily identify changes in the Track Changes document, edited portions of the text are annotated with a red-outlined box, labeled by comment number (e.g., R3.1). Our responses to each reviewer comment are below in italics.

Comments:

R3.1 Authors have addressed all the comments satisfactorily. However, the concentration of cytokine presented in Supplemental Figure 3 and Supplemental table 3 do not match. In figure all the cytokine presented as ng/ml. The legends needs to be more informative and include all details.

Both S3 Figure and S4 Table present the cytokine concentrations in pg/mL. This detail has been made explicit in the caption for S3 Figure to be more informative. We have also checked that all supplemental information is clearly referred to within the main body of the text.

R3.2. In the supplemental table - authors should provide the average CT for the targets studied.

We have included an additional supplementary table (Table S3) that includes the average Cq value for each gene of interest for every group.

Thank you considering our revised manuscript.

Sincerely,

Deva D. Chan, Ph.D.

WELDON SCHOOL OF BIOMEDICAL ENGINEERING
Martin C. Jischke Hall of Biomedical Engineering
206 S. Martin Jischke Drive, West Lafayette, IN 47907
Email : chand@purdue.edu | Office: 765-496-1768
