# Supplementary material for: Endogenous production of hyaluronan, PRG4, and cytokines is sensitive to cyclic loading in synoviocytes
Source: PLoS One. 2022 Dec 28;17(12):e0267921. doi: 10.1371/journal.pone.0267921 (PMC9797074; doi:10.1371/journal.pone.0267921)
Supplement: Attachment — Submitted filename: Response to Reviewers Only.docx [file pone.0267921.s010.docx]

To the reviewers,

Thank you for the additional comments on our manuscript titled “Endogenous Production of Hyaluronan, PRG4, and Cytokines is Sensitive to Cyclic Loading in Synoviocytes.” Each point has been addressed and changes to the original manuscript are in a Track Changes version. To easily identify changes in the Track Changes document, edited portions of the text are annotated with a red-outlined box, labeled by comment number (e.g., R2.1). Our responses to each reviewer comment are below in italics, with quote blocks (**bolded**) for any portions of text that were substantially changed (changes are in **blue font**).

Comments:

1. Many of the cytokines Il1Ra and IL-1b are reported to be responsive to cyclic strains. However, these cytokines were out of the standard curve in the current study. Are these cytokines falling above or below standard curve? If it is above, why was dilution not considered for estimating the concentration?

*R2.1. Cytokines that were not included in the analysis, including IL1-Ra and IL-1b, fell below the standard curve. This detail has been updated in the text:*

**Cytokines whose concentrations were out of range fell below the lower limit for all samples and were excluded from analyses.**

2. mRNA expression of these cytokines could also have been studied.

*R2.2. Indeed, gene expression of these cytokines could have been measured; however, this study was focused on the availability of these cytokines in the media, enabling inference of cytokines that may be made available in the joint space. Gene expression related to a particular cytokine would have been directly linked to its protein expression, which is the end product that is made available into the media. On the other hand, we opted to measure gene expression of hyaluronan-related genes because multiple gene products contribute to and affect hyaluronan content in the media, with multiple degrees of separation between gene expression and product availability. In the case of hyaluronan availability, while differential expression of Has1-3 genes may be linked to their respective protein (HAS1-3) expression levels, the activity level of these synthase proteins is what directly adds to available hyaluronan.*

3. Since synoviocytes cell line (tumor cells) was used – cyclic strains are density dependent - how was cell density optimized for the study?

*R2.3. Cell density was optimized to balance adhesion of cells to the silicone membrane with total cells stimulated in each well. Through pilot experiments, we observed that high confluency caused detachment of cell monolayers from the silicone membrane. To maintain attachment, confluency was therefore limited to 70-80%. Specifically, in pilot studies, we varied seeding density between 2x10^4^ and 5x10^4^ cells/mL and found that concentrations above 4x10^4^ cell/mL resulted in detached of the cell layer. 2x10^4^ cells/mL was not enough to isolate enough total RNA without surpassing the number of wells that could be considered technical replicates and therefore pooled for analysis. Therefore, our final seeding density of 3.5x10^5^ cells/mL (3-4x10^4^ cells/cm^2^) was used. Each well was plated after confirmation of cell numbers using a cell counter. We have added the following to the text to clarify the results of the pilot studies in guiding these ecperiments:*

**SW982 cells were seeded on silicone membranes (BioFlex plates, Flexcell International Corp, Burlington, NC) at a density of 3-4 × 10^4^ cells/cm^2^ (optimized during pilot experiments to minimize cell detachment while preserving adequate total RNA yield) and were allowed to attach for 24 hours in 1 mL complete medium.**

4. Please provide the table with the concentration of various cytokines (minimum, maximum). The cytokine is expressed ng or pg/ul rather than normalized to protein concentration. Did 10% or 20% cyclic strains induce cell death?

*R2.4. We measured protein concentration in each sample and found no significant differences between groups. A table with raw cytokine concentrations is included in Supplementary Table 3 for the interested reader, and complete data of both protein and cytokine concentration are available in our data repository. The supplementary figure shows units as pg/mL (which is the concentration of all standards for each cytokine).*

*While we did not directly quantify cell death, qualitative assessments throughout the study did not indicate cell death. Notably, previous studies that used cyclically loaded synovial fibroblasts to 10% strain have shown no changes to viability {Bader, 2010,* Cytokine*}.*

Thank you considering our revised manuscript.

Sincerely,

Deva D. Chan, Ph.D.

Assistant Professor

Weldon School of Biomedical Engineering

Purdue University

Assistant Professor (Adjunct)

Department of Biomedical Engineering

Rensselaer Polytechnic Institute
